# Coordination of Cell Polarity during *Xenopus* Gastrulation

**DOI:** 10.1371/journal.pone.0001600

**Published:** 2008-02-13

**Authors:** Asako Shindo, Takamasa S. Yamamoto, Naoto Ueno

**Affiliations:** 1 Division for Morphogenesis, Department of Developmental Biology, National Institute for Basic Biology, Myodaiji, Okazaki, Aichi, Japan; 2 Department of Basic Biology, School of Life Science, The Graduate University of Advanced Studies (SOKENDAI), Myodaiji, Okazaki, Aichi, Japan; Max Planck Institute of Molecular Cell Biology and Genetics, Germany

## Abstract

Cell polarity is an essential feature of animal cells contributing to morphogenesis. During *Xenopus* gastrulation, it is known that chordamesoderm cells are polarized and intercalate each other allowing anterior-posterior elongation of the embryo proper by convergent extension (CE). Although it is well known that the cellular protrusions at both ends of polarized cells exert tractive force for intercalation and that PCP pathway is known to be essential for the cell polarity, little is known about what triggers the cell polarization and what the polarization causes to control intracellular events enabling the intercalation that leads to the CE. In our research, we used EB3 (end-binding 3), a member of +TIPs that bind to the plus end of microtubule (MT), to visualize the intracellular polarity of chordamesoderm cells during CE to investigate the trigger of the establishment of cell polarity. We found that EB3 movement is polarized in chordamesoderm cells and that the notochord-somite tissue boundary plays an essential role in generating the cell polarity. This polarity was generated before the change of cell morphology and the polarized movement of EB3 in chordamesoderm cells was also observed near the boundary between the chordamesoderm tissue and naïve ectoderm tissue or lateral mesoderm tissues induced by a low concentration of nodal mRNA. These suggest that definitive tissue separation established by the distinct levels of nodal signaling is essential for the chordamesodermal cells to acquire mediolateral cell polarity.

## Introduction

Cell polarity is essential for embryogenesis that involves asymmetric cell division, cellular morphogenesis, directed cell migration, and remodeling of tissues. Cell polarity also generates functional biases within a cell by properly localizing signaling components and cellular structures such as cell protrusions. Therefore, disruption of cell polarity results in abnormal cell behavior and is known to cause congenital anomalies.

During *Xenopus* gastrulation, the embryo elongates along an anterior-posterior (AP) axis, largely due to a cell movement called convergent extension (CE) in which cells are polarized and change their morphology to spindle-shape and intercalate each other [1]–[3]. This mediolateral intercalation is converted to the force to elongate the mesodermal cell mass antero-posteriorly and is believed to be the driving force to elongate the embryo proper from its spherical shape. It is well known that during CE the mesoderm cells display polarized morphology is associated with drastic changes in cytoskeletal arrangement [4]. The morphological as well as functional polarization is manifested by the restricted localization of cellular protrusions as well as other functional components such as dishevelled, actin [5], and XGAP [6] at both ends of the spindle-shaped cells and this biased localization of signaling proteins leads to the formation of cell protrusions at the ends of the cells.

Recent studies revealed that the cellular polarization is controlled by a system similar to planar cell polarity (PCP) pathway initially described in *Drosophila* as an essential pathway for wing epithelium to set up cell polarity directing distally oriented wing hair and organized photoreceptor cells in ommatidium [7]. It is now well established that vertebrates co-opted the pathway to regulate the cell polarity of mesodermal cells contributing to gastrulation, and to orient hair cells in the inner ear [8]. To date, a number of signaling components acting in the pathway have been identified. They include Frizzled, Strabismus/Van goch, Flamingo, Prickle, Diego, etc [9]. Furthermore, in addition to the core PCP pathway components, a number of molecules have been identified as essential components for cellular morphogenesis prerequisite to CE [10], [11]. These suggest that sequential and/or parallel events that take place following the initial establishment of functional polarity along the mediolateral axis are essential for the cells to pull each other with the protrusions, intercalate each other, and eventually converge. However, molecular and cellular understanding as to when the polarity is established and how it is conferred on the cells remains unclear.

We reasoned that one of the difficulties hampering the understanding of this process comes from a shortage of methods to evaluate the functional cell polarity, which is observable as intracellular events. In mammalian cultured cells, it is well known that microtubules (MTs) are involved in defining the cell polarity [12], [13] and showing functional polarity (i.e. migrating cells, nerve cells, epithelial cells) [14], [15]. Furthermore, it has also been reported that MTs are required for polarization cells that undergo mediolateral intercalation during gastrulation [16]. In this study, using *Xenopus laevis* as a model, we therefore attempted to visualize MT dynamics by using EB3 (end binding 3) [17] proteins that bind the ends of MTs as the indicator of the intracellular events during convergent extension, and attempted to correlate it with the establishment of cell polarity. As a result, we found that the early notochord-somite boundary has information essential for triggering cell polarity of the mesodermal cells. We also found that physical contact with tissues with different cell properties mimic the role of the boundary. From these results, we propose the possibility that the information for cell polarization might be generated by the definitive tissue separation.

## Result

### The tracking of EB3-GFP movement reveals microtubule growth in Xenopus explants

To investigate whether the MT dynamics in chordamesoderm cells are polarized, we observed and analyzed the EB3-GFP movement, and compared them in the chordamesoderm cells of Keller explants and ectoderm cells of animal caps.

**Figure 1:**
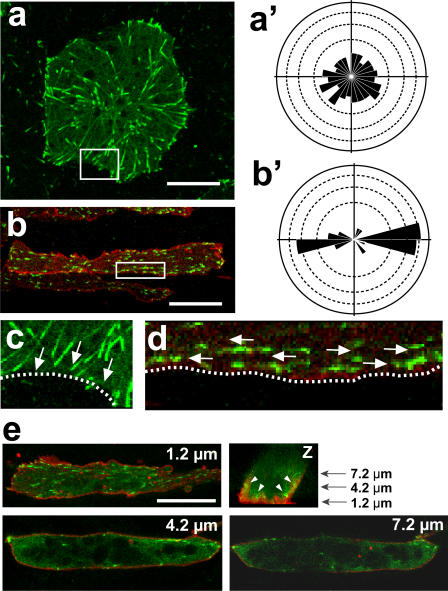
Tracking of EB3-GFP movement shows microtubule growth in Xenopus explants. (a) In an animal cap cell, EB3-GFP molecules show radially symmetrical movement toward the rim of the cell in the rose diagram (a'). (b) In a chordamesoderm cell, the movement was mostly bidirectional toward both ends of the cell (b'). (c, d) Magnification of the membrane region (white box) of a and b shows that EB3 moved toward the cytoplasmic membrane and disappeared after attaching to it in an animal cap cell (c), while EB3 moved along the membrane in a dorsal mesoderm cell. Arrows show the direction of the EB3 movement, and dotted lines indicate the cytoplasmic membrane. (d). (e) Z section of chordamesoderm cell reveals that most of the EB3 comets are observed in the plane, which is 1.2 µm from glass dish. In the planes, which are 4.2 µm and 7.2 µm from glass dish, the yolk granule were located in the center region of the cell and EB3 comets were located only near the cytoplasmic membrane (white arrow heads). Scale bars, 20 µm. Each scale of the rose diagrams corresponds to 10%.

In animal cap cells, the EB3 fluorescence migrated from various points toward the rim of the cell in a radially symmetrical manner (Fig. 1a, a', Movie S1). In contrast, in Keller explants, specifically in the spindle-shaped chordamesodermal cells that are fated to undergo convergent extension, the EB3-GFP moved toward both tips of the cells (Fig. 1b, b', Movie S2). Interestingly, a detailed analysis demonstrated that in sharp contrast to animal cap cells, the EB3 movement in Keller explant cells was exclusively in the mediolateral direction in any area of the cell, and the MT plus ends seemed to interact with the cell cortex only at the two cell tips (Fig. 1c, d, Movie S3). A three-dimensional analysis showed that the most of the EB3 comets were abundant in the planes proximate to the fibronectin-coated dish (1.2 µm in Fig. 1e, Movie S4), but fewer in upper planes (4.2 µm and 7.2 µm in Fig. 1e). These results suggest that MT growth takes place dominantly at the bottom but not middle region of the cells in this condition and that our observation of the bottom plane faithfully reflects overall polarity of MT growth.

Taken together, these results showed that the highly polarized cells in the explants also displayed polarized MT growth, and demonstrated that the visualization of MT growth orientation by tracking EB3 movement was a useful method for evaluating functional planar cell polarity.

### The notochord-somite boundary contains the factors that attract the EB3 movement

**Figure 2:**
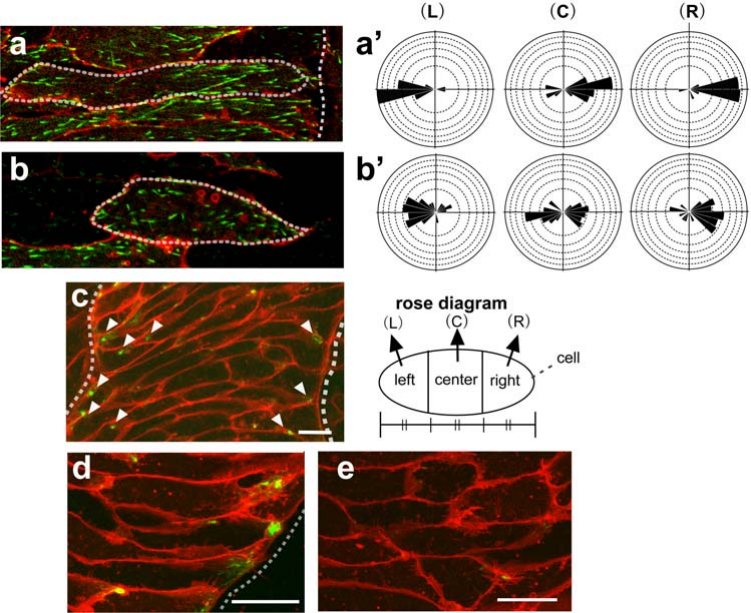
Notochord-somite boundary and/or extra-notochord tissue attracts EB3 movement. (a) A relatively late notochord cell (Stage 13∼) that was proximal to the boundary. The cell was divided into three regions (left: L, center: C, right: R) of equal horizontal (mediolateral) length for tracking the EB3 comets to clarify the direction of their movement. The EB3 movements were highly biased and mostly rightward in the center portion (a'). (b) A late notochord cell (Stage 13∼) that was distant from the boundary. No significant bias of the EB3 movement was observed (b'). (c) APC-GFP localization in the notochord in Keller explant. (d) APC-GFP localization in the chordamesoderm cells near the boundary in Keller explants. The localization was restricted to the one cell end pointing toward the boundary. (e) APC-GFP localization in the chordamesoderm cells located in the center of the notochord. Note that the localization was diffuse and unfocused. The dotted line indicates the notochord-somite boundary, and arrowheads indicate the localization of APC-GFP. Scale bars, 20 µm. Each scale of the rose diagrams corresponds to 10%.

The most intriguing observation from the Keller explants was that, in the cells close to the notochord boundary, EB3 preferentially moved toward the boundary. This suggested that MT growth in embryonic cells is a regulated process that is controlled by extracellular stimuli. To confirm the effect of the tissue boundary on the direction of EB3 movement, we carefully observed the EB3 movement in chordamesoderm cells in different positions relative to the notochord, when intercalation was almost complete. We specifically focused on cells that were in contact with the notochord-somite boundary and those that were in the middle of the notochord. Confirming our initial observation, we found that EB3 had a strong tendency to move toward the notochord-somite boundary in cells that were in contact with the boundary. For example, in Fig. 2, in the cell whose right (lateral) side touches the boundary, most of the EB3 comets moved toward the boundary (Fig. 2a, Movie S5). The farther away from the boundary the cells were, the less obvious was the directional bias of the EB3 movement. In cells not touching the boundary, i.e., located in the center of the notochord, EB3 moved more randomly and did not show biased movement, even at the same stage (Fig. 2b Movie S6). These results show that EB3 moved in an essentially monopolar manner toward the notochord boundary in cells near the boundary. The tracking analysis was performed for three different regions of the cell (left, center, and right), and showed that most of the EB3 puncta originating from and passing through the center region, also moved toward the boundary side. Microtubule growth is thus strongly biased toward the notochord-somite boundary in notochord cells near the boundary, revealing one of the mechanisms for attraction.

We next examined the localization of Adenomatous poliposis coli (APC) [18], which is known in mammalian cells to anchor polarized MTs to the cell cortex and stabilize MTs [19]–[21]. In the presumptive notochord cells in Keller explants, APC was sharply restricted to cell tips facing the boundary (Fig. 2c, d). This localization was mostly observed in the cells near the boundary (Fig. 2d), and cells located at the center region of the notochord lacking the contact with the boundary, did not show polarized APC localization (Fig. 2e), which is consistent with the polarity of EB3. Furthermore, APC was either randomly localized or undetectable in normal animal cap cells (data not shown). These results suggest that mesoderm differentiation is a prerequisite for the monopolarized APC localization as well as the biased MT elongation and that the notochord boundary has critical information for the polarization.

### EB3 shows polarized movement independent of cell morphology in early convergent extension

**Figure 3:**
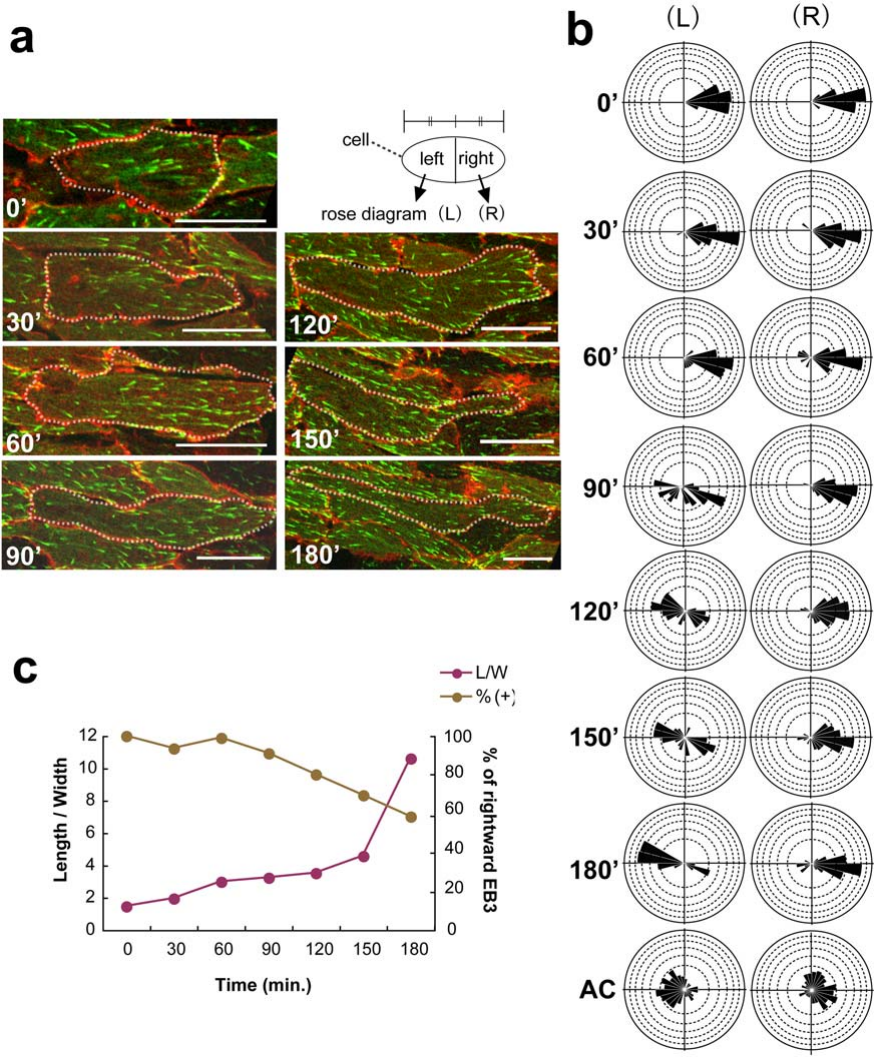
EB3 shows polarized movement independent of cell morphology in the early phases. (a) Time course of cell-shape change from 0' to 180' in Keller explants. The dashed lines outline the shape of the cells. (b) Rose diagrams of EB3 movement in the indicated cells. Each cell was divided into left and right portions at the center of the horizontal (mediolateral) cell length, and the EB3 movements in each portion were plotted as rose diagrams. (L) Left and (R) right. (c) Correlation between cell shape (length/width) and bias of EB3 movement (rightward EB3; −45° to +45°). Note the biased rightward movement without significant cell-shape change. Scale bars, 20 µm. Each scale of the rose diagrams corresponds to 10%.

We next examined the presumptive notochord cells in Keller explants during the early phase of CE, by observing both the cell morphology and EB3 movements over time, to determine when the polarization is initially set up. Around the onset of morphological polarization, we observed the EB3 movement by real-time confocal microscopy. We found that EB3 often showed uni-directional movement even before cell elongation (Fig. 3a, Movie S7). The polarization prior to the morphological change was most evident in cells adjacent to or near the notochord-somite boundary (Fig. 2). For quantification, the observed cells were divided into two regions (left, right) of equal mediolateral length, and the direction of EB3 movement in each region was plotted (Fig. 3b-L, -R). The results clearly showed that the EB3 movement was highly polarized even before the cell morphology changed in the presumptive notochord cells, whereas in control animal cap cells it was rather random. We confirmed these finding by measuring EB3 movements in several cells and obtaining essentially the same quantification results (data not shown). Once the morphological polarization and intercalation of the cells had become more evident, the EB3 movement in the boundary-side region (R) became highly polarized, as described above, and, with a slight delay, movement toward the opposite tip of the cell (L) also became visible. In sharp contrast, EB3 movement was not polarized in animal cap cells, regardless of their morphology (data not shown). We next examined the possible correlation between cell morphology and polarized EB3 movements and found that at time 0' when no obvious morphological change of cell shape is detected, the cells show biased movements of EB3 (Fig. 3c).

These results clearly show that the planar polarity revealed by the EB3 movement is established at an early phase, even before the cells undergo morphological change. This result also shows that the functional polarity revealed by the growth direction of MTs is different from the morphological polarity that is reflected in the cell shape.

### Animal cap cell polarity requires induction of chordamesoderm and the reception of signals from an adjacent explant with distinct properties

We next examined the predicted attractive cue(s) in the notochord boundary or in the somite, using activin- or nodal-expressing animal caps. We used animal caps for these experiments, because Keller explants are already fated to be notochord and extra-notochord tissue, and are therefore likely to contain various secreted factors that attract EB3 movement. It was previously reported that a high concentration of activin / nodal induces notochord differentiation in animal caps, and that lower concentrations induce lateral mesoderm [22], [23]. We therefore prepared animal caps in which chordamesoderm was artificially induced by a high concentration of nodal (125–150 pg: nodal-expressing animal cap (NAC)), in which the cells are barely polarized, and co-cultured them with animal caps representing other types of tissue, including extra-chordamesoderm (such as peri-chordamesoderm, ectoderm, or lateral mesoderm) (Fig. S1), and investigated which tissue was capable of attracting the EB3 movement in chordamesoderm cells.

**Figure 4:**
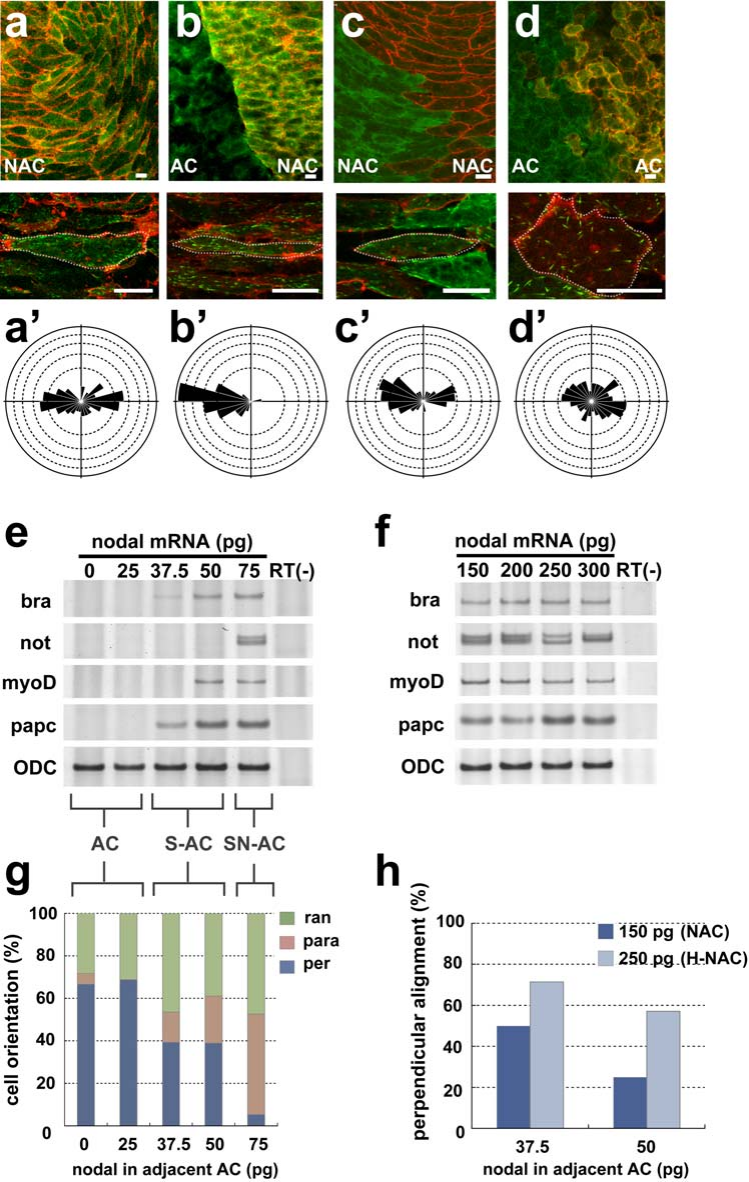
A heterogeneous combination of tissues can confer planar cell polarity. (a) The single culture of animal cap expressing a high concentration of Nodal mRNA (125–150 pg: NAC). (b) Combined culture of uninjected animal cap (AC) and NAC. Homogenous combined cultures of NAC (c) and AC (d). The EB3 in the cells in panels a–d respectively, was tracked. (a'–d') Rose diagrams of the EB3 movements in cells a–d. (e) RT-PCR (reverse transcription-polymerase chain reaction) analysis of low concentration of Nodal-mRNA (25–75 pg) injected animal caps. Animal caps into which a relatively high concentration of Nodal mRNA (75 pg: SN-AC) was injected expressed the chordamesoderm and somite marker, Xnot and XmyoDa, papc, and those receiving a relatively low concentration of Nodal mRNA (37.5–50 pg: S-AC) expressed only the somite marker, XmyoD. (f) RT-PCR analysis of high concentration of Nodal-mRNA (150 pg–300 pg: NAC, H-NAC). (g) The angle of the cell body in relation to the border in NAC (150 pg) combined with explants treated with different Nodal levels (0–75 pg of mRNA). Cell alignment perpendicular to the boundary, representing the mediolateral polarity, was observed when the combination was heterogeneous and loss when the combination became more homogeneous (37.5–75 pg). (per: perpendicular, para: parallel, ran: random) (h) The ratio of perpendicularly aligned cells in relation to the border was altered by difference of nodal signaling level. The cells in H-NAC (250 pg) tended to align perpendicular to the border with S-AC (37.5–50 pg). Scale bars, 20 mm (a–d). Each scale of the rose diagrams corresponds to 10%.

Despite the spindle-shaped morphology of the NAC cells, they showed non-biased EB3 movement when they were cultured alone (Fig. 4a, Movie S8), suggesting that chordamesoderm differentiation is required but not sufficient for the cells to acquire functional cell polarity. On the other hand, when the NAC was co-cultured side-by-side with normal animal cap (ectoderm), the EB3 in the NAC cells moved toward the boundary between the two explants with strikingly biased movement (Fig. 4b), reminiscent of the boundary-contacting cells in the Keller explants (Fig. 2a). The biased EB3 movement was absent when NAC was cultured with NAC, or when animal cap was cultured in conjunction with animal cap (Fig. 4c, d). We also found that the disruption of the biased EB3 movement in NAC correlated well with aberrant cell alignment in the tissues. When NAC was conjugated with animal cap, the polarized cells were aligned perpendicular to the boundary, as described above (Fig. 4g). We also tested the conjugation of NAC and animal cap cells injected with increasing concentrations of nodal mRNA (25–75 pg). The lowest concentrations (0–25 pg) induced neither somite nor notochord markers (animal cap: AC), and modest concentrations (37.5–50 pg) only induced somite markers (S-AC). Higher concentrations (75 pg) of nodal mRNA induced both somite and notochord markers (SN-AC) (Fig. 4e). When NAC was conjugated with S-AC, we unexpectedly found that the biased EB3 movement was less evident, and the ratio of the perpendicularly aligned cells representing notochord cells in vivo rather decreased (Fig. 4g). Very interestingly, however, when nodal concentration in NAC was increased even higher (200–300 pg: H-NAC) to ensure the notochord differentiation, cells aligned perpendicularly to the boundary were restored (Fig. 4h, f). These results strongly suggest that distinct nodal signaling levels, which in turn establish different populations of cells and hence inter-tissue boundaries, provide a cue for the cell polarity and cell alignment. In addition, cells in NAC (150 pg) combined with SN-AC tended to elongate in parallel to the tissue boundary (Fig. 4g), which is consistent with the previous finding that cells are polarized mediolaterally by sensing a shallow activin gradient generated along A-P axis [24]. These findings further suggest that mediolateral polarity is established in notochord cells by the cells' detection of a difference in nodal signaling, and that this polarity is essential for the notochord cells to be anchored to the boundary with non-notochord cells and aligned vertical to the AP axis.

We next confirmed that the effect of nodal is mediated by Smad2. The conjugation of Smad2-expressing animal cap and normal animal cap indicated that the influence from the tissue boundary was observed only in the chordamesoderm cells expressing Smad2. The results showing that the EB3 in animal cap did not respond in this co-culture system again suggest that chordamesoderm cells but not animal cap cells are capable to respond to the predicted polarity cue(s) (Fig. S2).

## Discussion

By tracking EB3-GFP as an indicator of functional polarity, we have been able to visualize one of the intracellular events in mesoderm cells during CE. We found that imaging EB3-GFP movement in explants by a confocal microscope gives a sufficient sensitivity despite autofluorescence of yolk granules and time- and space resolution to monitor MT growth in a single cell during cell morphogenesis and intermingling, and proved that the construct is useful to evaluate in real time the functional cell polarity being established, as has been shown with *Drosophila* [25] and mammalian cultured cells [17]. The most significant though somewhat unexpected finding in the present work is that chordamesoderm cells are polarized and elongated in a rather unidirectional manner, preferentially directed to the notochord-somite boundary even before cells changed their morphology. In previous studies, the mesoderm cells contributing to gastrulation were thought to have a bipolar nature mainly based on their morphology: symmetrically elongated and spindle-shaped cells manifested a typical bipolarity. Furthermore, we demonstrated that several polarity proteins such as aPKC and PAR proteins are localized to the two ends of the polarized cells [6]. We also predicted that proteins are localized to both ends of these cells via bi-directional, kinesin motor protein (KIF)-mediated transport along MT tracks, as demonstrated for PAR-3 in neurons [26]. In this study, however, we observed a strong mono-polarity of MTs particularly in the cells close to the notochord boundary. We reason that cells in the central region of the notochord may be weakly polarized, and therefore showed rather undirected EB3 movement. In other words, cells located away from the boundary may be less influenced by the polarity cue(s). This view is strongly supported by the mono-polar localization of APC in only a few layers of cells proximate to the boundary.

In mammalian cells, APC is shown to be required for the parallel array of MTs [27]. In addition, disruption of APC resulted in the loss of cell migratory activity. On the contrary, overexpression of APC promotes the formation of cell protrusions [28]. Although it remains unclear whether APC directly dictates the cell polarity, it may contribute to the establishment of the polarity through the stabilization of MTs. In spite of the function of APC in the cell polarity and CE needs to be clarified further, our finding of the polarized localization of APC may provide a clue to understand the intracellular mechanism of the cell polarity formation.

We also found that physical contact with tissues with different cell properties can mimic the role of the boundary for biased EB3 movements, and the interaction with juxtaposing tissues is important for chordamesoderm cells to acquire mediolateral polarity. In addition, we demonstrated that the clear difference of nodal signaling between the two tissues were necessary for the biased EB3 and coordinated cell alignments according to the tissue boundary. It is possible that in vivo this polarization is mediated by the interaction of definitive chordamesoderm and somite juxtaposed with each other. It is not clear, however, what actually mediates this process. Although one might speculate that somitic tissue secretes some factors to trigger cell polarity formation in chordamesoderm, it may be unlikely because even agarose gel juxtaposed to chordamesoderm could confer the cell polarity to the tissue (data not shown). Alternatively, this inter-tissue communication might be produced by the interaction of two cell populations with different adherent properties such that cells failed to adhere with different tissues propagate a cue for the polarity.

It is also possible that extracellular matrices (ECMs) deposited between the in vivo notochord-somite boundary as well as in the experimental boundary between notochord and may act as a polarizing cue. We therefore investigated the possible role of an ECM protein fibrillin [29], [30], a likely candidate for the polarizing signal, localized at the notochord-somite boundary. However, the expression of fibrillin protein at the notochord-somite boundary was relatively late (Fig. S3) and thus it is unlikely to be involved in the establishment of mesodermal cell polarity revealed by the EB3 movement. Nevertheless, we found that MTs were necessary for the accumulation of fibrillin to the boundary (Fig. S3). This suggests that accumulation of fibrillin may be a consequence of the establishment of the cell polarity. Another important question is why only the definitive chordamesoderm but not the presumptive ectoderm can respond to the polarity cue. To this end, we also investigated the possible role of PCP pathway because the PCP pathway regulates the morphological polarity as well as the functional polarity of cells [31]. We disrupted the PCP pathway component Dishevelled and found that it was not necessary for the biased EB3 movement in activin expressing animal cap cells near the tissue boundary (Fig. S4), showing that the biased EB3 movement toward the boundary is independent of PCP pathway.

Despite these attempts to clarify the polarizing signal(s) and cell responsiveness, exactly how the actual triggering signal is generated between two different tissues and how the tissue differences are sensed currently remains unknown. The real-time imaging of EB3 movement after gene manipulations will be a valuable approach for elucidating these processes.

## Materials and Methods

### Embryo manipulations

*Xenopus* eggs were collected as described [32], and the embryos were staged according to Nieuwkoop and Faber [33]. Capped mRNAs were synthesized using the mMESSAGE mMACHINE SP6 kit (Ambion), and purified on a NICK column (Pharmacia, Uppsala, Sweden) before being injected into four-cell-stage embryos. Keller explants were excised at stage 10.5, then placed in 0.1% bovine serum albumin (BSA)/1× Steinberg's solution and mounted to glass dishes coated with fibronectin (FN) (0.5 mg/ml, F1141; Sigma-Aldrich) for the observation of CE by confocal microscopy.

### Constructs

Plasmids encoding GFP-fused EB3 and APC were gifts from Drs. Akhmanova and Mimori-Kiyosue, respectively. Each construct was digested with the appropriate restriction enzyme and inserted into pCS2+ or pCS2p+ plasmids. All the plasmids were linearized with NotI for transcription. Capped mRNAs were synthesized with the mMESSAGE mMACHINE kit (Ambion) and purified on a NICK column (Pharmacia).

### Live-color imaging and time-lapse confocal analysis of cell behavior

Live-color imaging of *Xenopus* embryos was carried out as described [34], with minor modifications. Briefly, 40–250 pg of EGFP- or RFP-tagged mRNA was injected into two- or four-cell-stage embryos. Keller explants were isolated from stage 10.5 embryos and cultured in 0.1% BSA/1× Steinberg's solution in a glass-bottomed dish coated with FN, at 13°C for 20–24 hr or at 22°C for 8–12 hr, with similar results. The observation was initiated just before the cells became spindle shaped, and was performed with a laser-scanning confocal microscope (Carl Zeiss LSM510). Time-lapse images of the EB3-GFP movement were recorded every 2 sec, and 35 stacked images were composed. The focal plane was 1-µm deep in the tissue from the finronectin-coated dish.

### Tracking and analysis of EB3 movement

EB3 movements in time-lapse images of 35 frames/70 sec were analyzed by the tracking tool (Track Points) of the MetaMorph software (Molecular Devices Corporation). The absolute angle for every two points (frames) of a single EB3 comet in motion in reference to the border was calculated, and the average angle of a single EB3 comet and the number of comets were evaluated with and are shown as rose diagrams (Rose 2.1.0). The rose diagrams show the normalized frequency of EB3 directions in the cells directed into 15°bins representing 360°around the starting point of each EB3 track. Rose 2.1.0 was obtained from (phd.indiana.edu/∼tthomps/programs/home.html).

### Conjugation assay

An activin- or nodal-expressing animal cap was cut out at stage 9, mounted onto a FN-coated glass dish, and a normal animal cap was mounted next to it as the adjacent tissue (Fig. S1). These explants were co-cultured in 0.1% BSA/1× Steinberg's solution. Membrane-RFP was co-injected with the activin- or nodal mRNA to distinguish them from normal animal cap.

### Reverse transcription-polymerase chain reaction (RT-PCR)

RT-PCR was carried out as reported [32]. The PCR primer sequences used for analysis of Xnot, Xpapc and ODC, an internal input control, were as previously described [35], [36] [http://www.hhmi.ucla.edu/derobertis/]. The expression of each molecular marker was detected by PCR using the following specific primers: XmyoDa, upstream, agctccaactgctccgacggcatga, and downstream, aggagagaatccagttgatggaaaca, Xbra, upstream, ggatcatcttctcagcgctgtgga, and downstream, gttgtcggctgccacaaagtcca. For the analysis, 7 animal cap explants injected nodal mRNA were detached at stage 9, and assayed when sibling embryos reached stage 15.

## Supporting Information

Figure S1 See Experimental Procedures, “conjugation assay”
(2.35 MB TIF)

Figure S2 Smad 2 is an intracellular component of activin/nodal signaling, thus smad2-expressing AC induced chordamesoderm tissue cell autonomously. RT-PCR analysis showed that AC receiving over 250 pg smad2 mRNA expressed the chordamesoderm marker. When the AC was co-cultured with Smad2-AC, EB3 in the AC cells moved randomly, unlike that in the AC cells co-cultured with Activin expressing AC, which moved toward the boundary in a mirror image. These results suggest that the boundary between two distinct tissues has information to attract the EB3 movement; moreover, the attracting cue is specific for chordamesoderm tissue, because the EB3 in the AC moved randomly near the tissue boundary.
(6.71 MB TIF)

Figure S3 (a) Fibrillin antibody staining of a Keller explant at Stage 12. Fibrillin was localized to the cytoplasm (puncta). (b) Stage 14. The fibrous structure of fibrillin was formed. (c) Stage 16. The dotted line shows the notochord-somite boundary. An enrichment of fibrillin accumulation was observed around the notochord. (a'–c') Fibrillin antibody staining of nocodazole treated Keller explants at Stage 12∼16. The fibrillin accumulation around the notochord was inhibited by nocodazole, an inhibitor of MT polymerization.
(4.26 MB TIF)

Figure S4 To investigate the relevance of the PCP pathway in the biased EB3 movement, a dominant-negative form of Dishevelled (D2) was co-injected with activin mRNA. The EB3 in D2-AAC moved toward the boundary with AC. This result shows that the biased EB3 movement is independent of the PCP pathway.
(3.25 MB TIF)

Movie S1 EB3 movement in animal cap cell. EB3-GFP molecules show radially symmetrical movement toward the rim of the cell.
(2.96 MB MOV)

Movie S2 EB3 movement in chordamesoderm cell. The EB3 movement was mostly bidirectional toward both ends of the cell.
(2.76 MB MOV)

Movie S3 Magnification of the membrane region. EB3 moved toward the cytoplasmic membrane and disappeared after attaching to it in an animal cap cell, while EB3 moved along the membrane in a dorsal mesoderm cell.
(2.39 MB MOV)

Movie S4 Z projection of the EB3 in chordamesoderm cell (Fig. 1e). The most of the EB3 comets were abundant in the planes proximate to the fibronectin-coated dish, but fewer in upper planes.
(12.29 MB MOV)

Movie S5 EB3 movement in the chordamesoderm cell touching the boundary. Most of the EB3 comets moved toward the boundary.
(3.12 MB MOV)

Movie S6 EB3 movement in the chordamesoderm cell that was distant from the boundary. EB3 moved more randomly and did not show biased movement.
(2.92 MB MOV)

Movie S7 Time course of cell-shape and EB3 movements from 0' to 180' in Keller explants. EB3 often showed uni-directional movement even before cell elongation.
(4.07 MB MOV)

Movie S8 EB3 movements in conjugation assay.
(3.38 MB MOV)
